# Ribonucleoprotein Therapy in Alzheimer's Disease?

**DOI:** 10.18632/aging.100672

**Published:** 2014-06-02

**Authors:** Je-Hyun Yoon, Myriam Gorospe

**Affiliations:** Laboratory of Genetics, National Institute on Aging-Intramural Research Program, NIH, Baltimore, MD 21224, USA

Age-related neurodegenerative pathologies like Alzheimer's and Parkinson's diseases are accelerated by a decline in neuronal homeostasis. In this issue of *Cell Reports*, Kang et al. [1] reports the post-transcriptional mechanisms that regulate the expression and processing of amyloid precursor protein (APP), a protein directly implicated in the pathogenesis of Alzheimer's disease (AD). Although much is known about APP processing and cleavage, the post-transcriptional steps controlling *APP* biosynthesis are poorly defined. The important advance by Kang et al. 2014 is discovering that a single RNA-binding protein (RBP), HuD, binds to *APP* mRNA and enhances its stability and translation in cells of neuronal origin, stabilizes *BACE1* mRNA, encoding the β-site APP-cleaving enzyme 1 (BACE1), and stabilizes BACE1 antisense (*BACE1AS*), a long noncoding (lnc) RNA that protects *BACE1* mRNA from degradation. The positive influence of HuD on these RNAs and the encoded proteins was documented in the brains of mice overexpressing HuD as well as in patients with Alzheimer's Disease, and was linked to the accumulation of the toxic APP cleavage product Aβ in these systems. The results underscore a novel function for HuD in neurodegeneration through its regulation of RNA targets controlling Aβ production.

Given its joint influence on three functionally linked RNAs (*APP* mRNA, *BACE1* mRNA, and *BACE1AS*), that contribute to generating the neurotoxic peptide Aβ, HuD emerges from the studies by Kang and colleagues as a single factor that promotes APP processing to Aβ. Such a coordinated influence on a cellular function by a single RBP represents an example of the post-transcriptional operon/regulon proposed by Keene and Tenenbaum [2].

Besides orchestrating Aβ production, HuD was previously shown to repress translation of another target transcript, the insulin (*INS*) mRNA [3]. Accordingly, HuD-overexpressing mice exhibited an impaired ability to respond to a glucose challenge [3], a hallmark of diabetes, another pathology that escalates with age. Given that the same RBP, HuD, is linked to neurodegeneration and to diabetes, there is growing interest in modulating HuD expression and/or function. The microRNA miR-375 represses HuD production by lowering the stability and translation of the mRNA that encodes HuD (*ELAVL4* mRNA) [4]. In this regard, the decreased β-cell mass exhibited by miR-375-null mice [5] is a provocative finding, although this phenotype has not yet been linked to the effects of miR-375 upon HuD in the pancreas. Whether miR-375-null mice have also displays increased HuD, APP, BACE1, *Bace1as*, and Aβ also remains to be studied. The microRNA miR-485, which lowers *BACE1* mRNA levels in competition with *BACE1AS*, is under-expressed in entorhinal cortex and hippocampus of Alzheimer's disease-affected individuals [6]. The stabilization of *BACE1AS* by HuD in Alzheimer's disease-affected individuals can further contribute to BACE1 expression. Whether these two microRNAs directly modulate the post-transcriptional operon governed by HuD in neurological (and possibly pancreatic) pathologies underscores their potential therapeutic interest.

At present there are no therapeutic interventions directed at HuD. A low-molecular-weight compound, MS-444, was developed to inhibit the function of HuR, a ubiquitous RBP related to HuD, aimed at suppressing cancer cell proliferation [7]. A similar strategy can be applied to designing low-molecular-weight chemicals directed at HuD, perhaps directed to its RNA recognition motifs (RRMs); this would permit a selective inhibition of HuD binding to AD targets [1] and to insulin mRNA [3]. Alternatively, strategies aimed at inhibiting PKC, a protein kinase responsible for enhancing neuronal ELAV (nELAV) levels [8], could reduce the expression of HuD in select individuals, although targeted inhibition of a broad-spectrum kinase such as PKC would pose important challenges. Targeted administration of miR-375 could also repress HuD expression, in turn reducing Alzheimer's disease-associated Aβ accumulation and/or restoring insulin production, although microRNA-based therapies also have severe limitations at present. Whether other conditions, such as the presence of high blood glucose, suppress the function of brain HuD (as observed for pancreatic HuD) awaits testing.

To develop effective interventions aimed at HuD, it is imperative that we first learn a great deal more about HuD – its RNA and protein ligands, its transcriptional and post-transcriptional regulators, as well as the enzymes and pathways that modulate HuD function post-translationally. We will then be better prepared to design HuD-suppressing therapeutics that might have a beneficial impact in the pathogenesis of two age-related conditions, Alzheimer's disease and diabetes.
